# Elevated AKR1C3 expression promotes prostate cancer cell survival and prostate cell-mediated endothelial cell tube formation: implications for prostate cancer progressioan

**DOI:** 10.1186/1471-2407-10-672

**Published:** 2010-12-06

**Authors:** Mikhail G Dozmorov, Joseph T Azzarello, Jonathan D Wren, Kar-Ming Fung, Qing Yang, Jeffrey S Davis, Robert E Hurst, Daniel J Culkin, Trevor M Penning, Hsueh-Kung Lin

**Affiliations:** 1 Arthritis & Clinical Immunology Program, Oklahoma Medical Research Foundation, 825 N.E. 13th Street, Oklahoma City, Oklahoma 73104, USA; 2 Department of Urology, University of Oklahoma Health Sciences Center, Oklahoma City, OK 73104, USA; 3 Department of Physiology, University of Oklahoma Health Sciences Center, Oklahoma City, OK 73104, USA; 4 Department of Pathology, University of Oklahoma Health Sciences Center, Oklahoma City, OK 73104, USA; 5 Oklahoma City Department of Veterans Affairs Medical Center, Oklahoma City, OK 73104, USA; 6 Department of Biochemistry and Molecular Biology, University of Oklahoma Health Sciences Center, Oklahoma City, OK 73104, USA; 7 Center of Excellence in Environmental Toxicology, Department of Pharmacology, University of Pennsylvania School of Medicine, Philadelphia, PA 19104, USA

## Abstract

**Background:**

Aldo-keto reductase (AKR) 1C family member 3 (AKR1C3), one of four identified human AKR1C enzymes, catalyzes steroid, prostaglandin, and xenobiotic metabolism. In the prostate, AKR1C3 is up-regulated in localized and advanced prostate adenocarcinoma, and is associated with prostate cancer (PCa) aggressiveness. Here we propose a novel pathological function of AKR1C3 in tumor angiogenesis and its potential role in promoting PCa progression.

**Methods:**

To recapitulate elevated AKR1C3 expression in cancerous prostate, the human PCa PC-3 cell line was stably transfected with an AKR1C3 expression construct to establish PC3-AKR1C3 transfectants. Microarray and bioinformatics analysis were performed to identify AKR1C3-mediated pathways of activation and their potential biological consequences in PC-3 cells. Western blot analysis, reverse transcription-polymerase chain reaction (RT-PCR), enzyme-linked immunosorbent assay (ELISA), and an *in vitro *Matrigel angiogenesis assays were applied to validate the pro-angiogenic activity of PC3-AKR1C3 transfectants identified by bioinformatics analysis.

**Results:**

Microarray and bioinformatics analysis suggested that overexpression of AKR1C3 in PC-3 cells modulates estrogen and androgen metabolism, activates insulin-like growth factor (IGF)-1 and Akt signaling pathways, as well as promotes tumor angiogenesis and aggressiveness. Levels of IGF-1 receptor (IGF-1R) and Akt activation as well as vascular endothelial growth factor (VEGF) expression and secretion were significantly elevated in PC3-AKR1C3 transfectants in comparison to PC3-mock transfectants. PC3-AKR1C3 transfectants also promoted endothelial cell (EC) tube formation on Matrigel as compared to the AKR1C3-negative parental PC-3 cells and PC3-mock transfectants. Pre-treatment of PC3-AKR1C3 transfectants with a selective IGF-1R kinase inhibitor (AG1024) or a non-selective phosphoinositide 3-kinases (PI3K) inhibitor (LY294002) abolished ability of the cells to promote EC tube formation.

**Conclusions:**

Bioinformatics analysis followed by functional genomics demonstrated that AKR1C3 overexpression promotes angiogenesis and aggressiveness of PC-3 cells. These results also suggest that AKR1C3-mediated tumor angiogenesis is regulated by estrogen and androgen metabolism with subsequent IGF-1R and Akt activation followed by VEGF expression in PCa cells.

## Background

The aldo-keto reductase (AKR) enzymes comprise a functionally diverse gene family [1]. Members of the AKR superfamily are generally monomeric (37 kD) cytosolic NAD(P)(H)-dependent oxidoreductases that convert carbonyl groups to primary or secondary alcohols, and share a common (α/β)_8_-barrel structural motif (visit: http://www.med.upenn.edu/akr) [2]. In humans, four AKR1C isoforms have been identified; they are known as AKR1C1 [20α(3α)-hydroxysteroid dehydrogenase (HSD)] [3], AKR1C2 (type 3 3α-HSD) [4,5], AKR1C3 (type 2 3α/type 5 17β-HSD) [6,7], and AKR1C4 (type 1 3α-HSD) [5]. Natural substrates for these enzymes include steroids [8,9], prostaglandins [10], and lipid aldehydes [11].

Originally cloned from human prostate [7] and placental cDNA libraries [12], AKR1C3 shares greater than 86% sequence identity with the other three highly related human AKR1Cs [13]. AKR1C3 catalyzes both androgen and estrogen metabolism. The relatively high 17β-HSD activity of this enzyme reduces Δ^4^-androstene-3,17-dione (Δ^4^-dione; a weak androgen) to yield testosterone (a potent androgen) [14], and reduces estrone (a weak estrogen) to yield 17β-estradiol (a potent estrogen) [13]. Using its 3α-HSD activity, AKR1C3 reduces 5α-dihydrotesterone (5α-DHT, a potent androgen) to 5α-androstane-3α,17β-diol (3α-diol, a weak androgen) [7]. As a result, AKR1C3 may be capable of modulating the amounts of potent androgen (testosterone and 5α-DHT) and estrogen (17β-estradiol) available for the androgen receptor (AR) and estrogen receptor (ER), respectively.

Deregulated AKR1C3 expression has been associated with multiple human cancers. Suppressed AKR1C3 expression has been demonstrated in breast cancer [15] and endometrial cancer [16], whereas elevated expression of this enzyme has been reported in squamous cell carcinoma of the head and neck [17]. In the prostate, low or undetectable levels of AKR1C3 are observed in normal prostate epithelium [18], whereas levels of AKR1C3 expression are significantly elevated in localized [19,20], advanced [21], and recurrent [22] prostate cancer (PCa).

Based on the observations that expression of AKR1C3 is elevated in both localized and metastatic PCa, the enzyme might modulate significant pathological activities in cancer development or progression. Studies so far have been focused on the potential androgenic effects mediated by AKR1C3 in the prostate; and elevated AKR1C3 expression in the prostate is thought to be responsible for testosterone and 5α-DHT accumulation and AR *trans*-activation in cancerous prostate [14,20,21]. In this report, an androgen insensitive, AR-negative human PCa PC-3 cell line was stably transfected with AKR1C3 cDNA to establish PC3-AKR1C3 transfectants and to recapitulate elevated expression of this enzyme in PCa. Microarray, bioinformatics, and literature analyses were used to explore possible pathological consequences of elevated AKR1C3 expression in PCa cells. In addition to elevated cell growth in PC3-AKR1C3 transfectants [23], AKR1C3 overexpression promoted angiogenic capability of PC-3 cells as evidenced by elevated levels of vascular endothelial growth factor (VEGF) expression and PC-3 cell-mediated endothelial cell (EC) tube formation on Matrigel. These results suggest that, AKR1C3-mediated steroid hormones or prostaglandin metabolism may promote aggressiveness of PCa through enhanced tumor angiogenesis.

## Methods

### Reagents and chemicals

PC-3 cells (CRL-1435) derived from a human bone metastatic tumor and LNCaP-FGC (LNCaP) cells (CRL-1740) were obtained from ATCC (Manassas, VA). An SV40 large T-antigen transformed human microvascular EC line, HMEC-1 [24], was generously provided by Dr. Michael Ihnat at the University of Oklahoma Health Sciences Center. RPMI 1640 medium, F-12 nutrient mix, MCDB 131 medium, penicillin-streptomycin, fetal bovine serum (FBS), pcDNA3 vector, Trizol, and Lipofectamine 2000 were purchased from Invitrogen (Carlsbad, CA). AG1042 and LY294002 were purchased from EMD Chemicals (Gibbstown, NJ). Matrigel basement membrane matrix was purchased from BD Biosciences (Bedford, MA). Mouse anti-AKR1C3 monoclonal antibody was produced in our laboratories [25]. Rabbit anti-insulin-like growth factor (IGF)-1 receptor (IGF-1R) β, rabbit anti-phospho-IGF-1R β (Tyr 1131), rabbit anti-Akt, and rabbit anti-phospho-Akt (Ser 473) polyclonal antibodies, as well as horseradish peroxidase (HRP)-linked goat anti-rabbit IgG were obtained from Cell Signaling Technology (Danvers, MA). Mouse anti-human β-actin antibody was obtained from Sigma-Aldrich (St. Louis, MO). HRP-conjugated goat anti-mouse IgG was obtained from KPL (Gaithersburg, MD). Bicinchoninic acid (BCA) protein assay kit and enhanced chemiluminescent (ECL) substrate reagent were obtained from Pierce (Rockford, IL). VEGF enzyme-linked immunosorbent assay (ELISA) kit was purchased from Ray Biotech (Norcross, GA).

### Prostate cell culture and transfection

PC-3 cells were cultured in complete growth medium consisting of F-12 nutrient mixture, 7% FBS, and 100 units/ml penicillin-100 µg/ml streptomycin. LNCaP cells were cultured in complete growth medium consisting of RPMI-1640, 10% FBS, and penicillin-streptomycin. HMEC-1 were maintained in MCDB 131 medium supplemented with 10% FBS and penicillin-streptomycin. All cells were maintained in a humidified cell incubator at 37°C and 5% CO_2_. AKR1C3 expression construct (pcDNA3-AKR1C3) was established as previously described [23]. Transfection of PC-3 cells with either pcDNA3 empty vector or pcDNA3-AKR1C3 construct was achieved using Lipofectamine 2000. Stable transfectants were established in the presence of 600 μg/ml G418 selection. PC-3 stable transfectants were designated as PC3-mock or PC3-AKR1C3 for cells transfected with pcDNA3 or pcDNA3-AKR1C3 plasmid, respectively. Multiple independent clones were isolated and expanded in the complete growth medium containing G418. PC3-AKR1C3 clones were confirmed with elevated AKR1C3 mRNA and protein expression as compared to parental PC-3 cells and PC3-mock transfectants. LNCaP-mock and LNCaP-AKR1C3 stable transfectants were also similarly established.

### RNA extraction and quality evaluation

Total RNA was isolated from 2 independent PC3-mock clones and 3 independent PC3-AKR1C3 clones for a comprehensive gene expression analysis using oligonucleotide arrays. Briefly, following PC-3 transfectants (5 × 10^5^) seeding and adherence, total RNA was isolated from these clones using Trizol followed by RNeasy^® ^Mini total RNA isolation kit (Qiagen, Valencia, CA) purification. Concentration of the isolated total RNA was determined with a Nanodrop scanning spectrophotometer; and quality of the total RNA was assessed using the ratio of 28:18 s rRNA by Agilent 2100 Bioanalyzer capillary gel electrophoresis system (Agilent Technologies, Santa Clara, CA).

### RNA labeling, microarray hybridization, and scanning

A total of 250 ng total RNA from each clone was labeled using the Illumina Total Prep RNA Amplification kit (Ambion, Austin,. TX). Briefly, cDNA was reverse transcribed from the total RNA after priming with T7-oligo(dT); and cRNA was synthesized from the T7 promoter in the presence of biotinylated UTP. Biotinylated cRNA was hybridized to Illumina HumanWG 6 v2 BeadChips containing a total of 48,702 probes (Illumina Inc., San Diego, CA). Hybridization was detected by incubating the microarray chips with streptavidin-Cy3 (Amersham Biosciences, Piscataway, NJ) and scanned on an Illumina BeadArray Reader.

### Data normalization and identification of AKR1C3 regulated genes

Raw expression values were first normalized based on a normal distribution of genes with low expression levels as previously described [26,27]. Briefly, normalization was performed for each array by first plotting a frequency histogram of the raw expression data for all genes. The histograms showed a right-skewed unimodal distribution curve with the mode around zero. A normal distribution curve representing random variability of the genes with low expression values was then fitted around the mode, mirroring Gaussian profile of the left part of the histogram. Two parameters [mean and standard deviation (SD)] were defined. The expression values then were normalized to the SD of the normal distribution after subtraction of the mean and then log_10_-transformed. The arrays were than adjusted to each other by robust linear regression [28,29]. Genes with expression values less than 3.0 (or 0.477 in log_10 _scale) were considered to be not expressed; this is equivalent of setting a threshold at 3 SD above noise level. Raw and normalized gene expression data are available on Gene Expression Omnibus (GEO accession GSE20956).

To identify genes that are differentially expressed between PC3-mock and PC3-AKR1C3 transfectants, an associative analysis was performed as described by Dozmorov *et al*. [30]. Briefly, in combined microarray datasets from both PC3-mock and PC3-AKR1C3 transfectants, a reference group of genes expressed above background with low variability of expression as compared with variability of all others was identified by a F-test. The variability in expression of these genes was assumed to represent the technical variability of the assay. Associative *t*-test (the standard *t*-test applied to the comparison of expression variability) was used to test the null hypothesis if a given gene under a given condition is associated with the reference group. Those with statistically significant variability over the reference set were assumed to represent variability due to the biological variables being manipulated.

An additional analysis was performed to minimize threshold choice effects in different statistical approaches in microarray data analysis [31]. Class comparison between PC3-mock and PC3-AKR1C3 transfectants was performed using the BRB ArrayTools. To use the BRB Array Tools, raw expression values were log_2 _transformed; and quantile normalization was applied. Differentially expressed genes between the two classes, PC3-mock and PC3-AKR1C3 transfectants, were identified by random-variance *t*-test. The random-variance *t*-test, implemented in the BRB ArrayTools, is an improvement of the standard *t*-test since it permits sharing information among genes with within-class variation, without assuming that all genes have the same variance [32]. A stringent significance threshold was applied to limit the number of false positive findings; genes were considered statistically significant if their *P*-values were less than 0.001. Lists of differentially expressed genes from associative *t*-test and class comparison analysis were compared; and genes identified by both analyses were selected for further analysis unless otherwise stated.

### Functional analysis and identification of canonical pathways

For pathway analysis of the microarray data, gene lists were analyzed by Ingenuity Pathway Analysis (IPA; Ingenuity^® ^Systems, Redwood City, CA, http://www.ingenuity.com), a web-based bioinformatics tool. Each gene list was tested against the full Illumina HumanWG 6 v2 dataset to identify significantly overrepresented functions and canonical pathways as compared to the same number of randomly selected genes. The most significant functions and canonical pathways with *P *< 0.05 were selected; and genes overrepresented in the functions and pathways were identified. The branches of pathways containing genes that are not expressed on microarrays were not considered functional.

### Analysis of 5'-flanking regions of the differentially expressed genes

To explore transcriptional regulatory mechanisms of the genes regulated in PC3-AKR1C3 versus PC3-mock transfectants, the promoter analysis and interaction network toolset (PAINT) http://www.dbi.tju.edu/dbi/tools/paint/) [33] was used to identify overrepresented *cis*-acting elements, or transcription factor binding sites, within the identified genes against the same transcription regulatory elements (TREs) selected randomly from the whole set of genes on the microarray. Statistical significance for testing the presence of TREs was set at *P *< 0.05; and additional filtering was performed by using a false discovery rate (FDR) as described in the text.

### Data validation by literature analysis

Literature-based matching of genes to concepts provides an advantage over gene ontology (GO) enrichment analysis in that first, GO annotation lags literature publications, and second, more importantly it provides a much broader matching of genes to concepts, such as diseases, phenotypes, chemical compounds, and other genes frequently associated with the gene set being analyzed. To perform literature analysis, lists of differentially expressed genes between PC3-mock and PC3-AKR1C3 transfectants were analyzed by a comprehensive, computational search of published MEDLINE abstracts *via *a software package called IRIDESCENT [34-36]. There were 7 and 39 up- and down-regulated genes in PC3-AKR1C3 transfectants, respectively, not mentioned in the literature and were automatically discarded, since, by definition, they could not share any literature-based commonalities with other genes. This left 56 up-regulated and 108 down-regulated genes available for analysis. IRIDESCENT uses a thesaurus of names and their synonyms obtained from public databases such as OMIM, Entrez Gene, GO, CHEMID, and Disease Ontology to recognize these "objects" and their common spelling variants (e.g., "IL2" and "IL-2") within text. All available MEDLINE records (approximately 18 million at the time of this writing) were processed to identify "objects" co-occurring within the same abstracts, and within the same sentences within those abstracts. The number of co-occurrences was tallied, with a weighting based on a previous study [35] of 0.5 for objects co-mentioned in the same abstract and 0.8 for objects co-mentioned in the same sentence, providing a network of terms connected by their relative strength of association. Once commonalities had been identified in the literature for a gene set, each was compared to an identically connected set within a random network model to calculate a ratio of how many connections were observed in the actual dataset versus how many connections would be expected by chance (Obs/Exp ratio), providing a measure of statistical enrichment for each term. The significance of enrichment was calculated using a Monte Carlo simulation and the associations reported were ≥ 3 SDs from the mean (*P *≤ 0.01).

### Western blot analysis

Western blot analysis was performed to confirm IGF-1R and Akt activation in PC3-AKR1C3 transfectants identified by bioinformatics analysis. Briefly, following cell (1 × 10^6^) seeding and adherence, PC3-mock and PC3-AKR1C3 transfectants were lysed with RIPA buffer supplemented with 5 mM EDTA, 0.1 mM phenylmethylsulphonylfluoride (PMSF), Complete Mini-Protease Inhibitor Cocktail, and PhosSTOP Phosphatase Inhibitor Cocktail (Roche Applied Science, Indianapolis, IN). Total cellular proteins were collected following centrifugation to remove cellular components and quantified using the BCA protein assay kit. Aliquots (30 µg) of the cellular proteins were separated on 10% Tris-HCl gels; and separated proteins were transferred onto PVDF membranes (Bio-Rad; Hercules, CA). The membranes were incubated with rabbit anti-human phospho-IGF-1R β (Tyr 1131) antibody (1:250), rabbit anti-human IGF-1R β antibody (1:1,000), rabbit anti-human phospho-Akt (Ser 473) antibody (1:250), or rabbit anti-human Akt antibody (1:1,500) followed by HRP-conjugated goat anti-rabbit IgG (1:2,000) incubation. Immunoreactive proteins were detected using the ECL reagent. The membranes were then stripped and incubated with a mouse anti-human β-actin monoclonal antibody (1:5,000) followed by HRP-conjugated goat anti-mouse IgG (1:10,000) incubation and ECL development.

### Quantification of VEGF expression

VEGF appears to be one of the most important factors released by tumor cells as a signal for angiogenesis. VEGF expression was first compared between PC3-AKR1C3 and PC3-mock transfectants. Levels of VEGF mRNA expression were determined in these transfectants using semi-quantitative reverse transcription-polymerase chain reaction (RT-PCR) method. Total RNA isolation, first-strand cDNA synthesis, PCR amplification using pre-designed VEGF isoform-specific primer pairs and pre-determined cycling conditions followed our reported procedures [37]. PCR amplified products were separated on 1% agarose gels and stained with ethidium bromide. Images of ethidium bromide-stained gels were acquired with Gel Doc 1000 (Bio-Rad) equipped with Quantity One^® ^imaging software (Bio-Rad). The involvement of IGF-1R and phosphoinositide 3-kinases (PI3K) in VEGF mRNA expression in PC3-AKR1C3 transfectants was studied by adding 20 µM AG1024 (IGF-1R selective tyrosine kinase inhibitor) and 10 µM LY294002 (non-selective PI3K inhibitor), respectively, to the cell cultures at 2 hours prior to total RNA isolation. The selected concentrations of AG1024 and LY294002 did not interfere with cell viability as determined by XTT assay (data not shown).

Total VEGF secretion by PC3-mock and PC3-AKR1C3 C1 transfectants was quantified in conditioned media using a RayBio^® ^human VEGF ELISA kit following the manufacture's recommendations. Briefly, 1 × 10^6 ^cells were seeded onto 60 mm cell culture plates in 2 ml completed medium in duplicate. At 24 hours following cell seeding, the conditioned media were removed and centrifuged at 300 × *g *to clear cellular components. Aliquots (100 μl) of the conditioned media were transferred to each well of the 96-well assay plate. Known quantities of VEGF A_165 _were added in separate wells, run in parallel, and used to construct a standard curve. The plate was read at 450 nm on a μQuant microplate reader (Bio-Tek Instrument, Winooski, VT). Results were normalized to total protein present in the media, as determined by the BCA assay, and reported as pg of total secreted VEGF/μg total protein.

### EC tube formation assay

An image-based, PCa cell-mediated EC tube formation on Matrigel assay was used as an *in vitro *model to assess enhanced angiogenic capability of PC3-AKR1C3 transfectants predicted by bioinformatics. Briefly, PC3-mock and PC3-AKR1C3 transfectants (8 × 10^4^) were re-suspended in 0.25 ml liquid Matrigel and plated into each well of 24-well tissue culture plates in duplicate. Following overnight incubation, 8 × 10^4 ^HMEC-1 in 0.25 ml of the MCDB 131 complete medium were introduced on top of the solidified PC-3 transfectant-Matrigel suspension. The co-cultures on Matrigel were monitored and photographed at 6, 12, 24, 48, and 72 hours following HMEC-1 cell addition using an Olympus IX51 inverted microscope equipped with SPOT Insight CCD digital camera and SPOT Advance software (Diagnostic Instruments, Sterling Heights, MI). Three images were randomly taken from each well. Images in JPEG format were uploaded into Image Pro-Plus 6 (Media Cybernetics, Bethesda, MD), converted to green on the RGB color channel to eliminate the red color of Matrigel, and flattened. The number of tubes was counted using the dendrite/tube template in the Image Pro-Plus software; and non-tube forming cells suspended on Matrigel were mathematically excluded from counting [37]. The co-culture HMEC-1 tube formation model was also applied to 2 clones of each from LNCaP-mock and LNCaP-AKR1C3 transfectants.

To determine the roles of IGF-1R and PI3K in prostate cell-mediated EC tube formation, parental PC-3 cells as well as PC3-mock and PC3-AKR1C3 transfectants were first incubated with 20 µM AG1024 or 10 µM LY294002 at 37°C for 2 hours. Cells were then harvested, washed with phosphate-buffered saline (PBS), and suspended in Matrigel followed by HMEC-1 addition, image acquisition, and EC tube quantification using the above described procedures.

### Statistical analysis

Statistical differences between PC3-mock and PC3-AKR1C3 stable transfectants were analyzed using student *t*-test. Statistically significant difference was set when *P *< 0.05.

## Results

### Identification of genes that are regulated by elevated AKR1C3 expression in PC3-AKR1C3 transfectants

Gene expression profiles were compared between PC3-mock and PC3-AKR1C3 transfectants using microarray and bioinformatics techniques to predict possible alterations of gene expression in AKR1C3-positive PCa. When a threshold was set at 3 SDs above noise level, approximately 12,000 genes were expressed above the level in all arrays. The BRB ArrayTools class comparison identified 70 genes being up-regulated and 153 genes being down-regulated in PC3-AKR1C3 versus PC3-mock transfectants. The associative analysis identified 27 genes being up-regulated and 85 genes being down-regulated by at least 1.5 fold and expressed greater than 20 SDs above the noise levels in PC3-AKR1C3 versus PC3-mock transfectants (Additional file 1). Class comparison and associative analysis provided almost overlapping results. The 27 up-regulated genes identified by the associative analysis were a subset of the 70 genes identified by the BRB ArrayTools. Among the 85 down-regulated genes identified by the associative analysis, 76 genes were among the 153 genes identified by the BRB ArrayTools (Additional file 2). The 27 up-regulated and 76 down-regulated genes in PC3-AKR1C3 transfectants identified by both analyses were used as "beacon" genes for further analysis to reduce the possibility of including false positives. However, in some cases, the number of "beacon" genes was insufficient to identify their common functions or other properties. Each "beacon" gene may represent a unique function or feature, and the statistical calculations for overrepresentation of such features become impossible. In such cases, the 70 up- and 153 down-regulated gene lists identified by the BRB ArrayTools were used, as described in the text. The inclusion of these genes was further validated by subsequent statistical analysis for overrepresentation of common features among genes in the lists.

### AKR1C3 effects on steroid signaling in PC-3 cells

**Figure 1:**
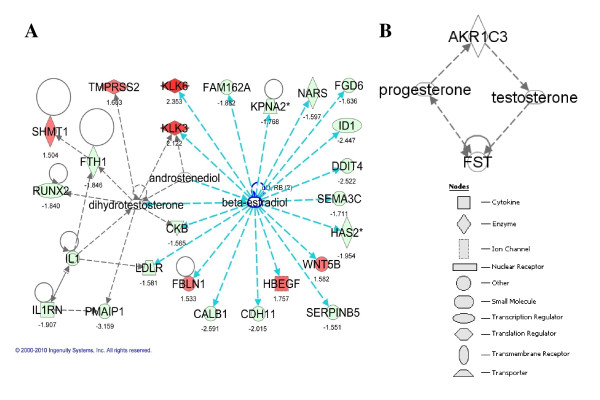
**Influence of steroid hormone signaling in PC3-AKR1C3 transfectants**. (A) Genes whose expression is altered in PC3-AKR1C3 transfectants were identified to be regulated by 17β-estradiol, androstenediol, or 5α-DHT. Genes that are labeled in red and green represent up- and down-regulated genes, respectively, in PC3-AKR1C3 transfectants as compared to PC3-mock transfectants. The fold changes are reflected by corresponding numbers under the gene names. (B) FST expression can be regulated by AKR1C3-mediated testosterone and/or progesterone metabolism in PC3-AKR1C3 transfectants. The legend for the gene shapes is at lower right corner.

Genes that are differentially expressed in PC3-AKR1C3 versus PC3-mock transfectants were submitted to IPA to predict pathways that are modulated by the AKR1C3-regulated genes. Manual inspection of pathways and networks assembled by Ingenuity suggested that AKR1C3 affected genes in estrogen and, potentially, androgen signaling pathways in PC-3 cells. A joint analysis of the 27 up- and 70 down-regulated genes ranked by ratios of gene expression between PC3-mock and PC3-AKR1C3 transfectants identified networks of genes mainly centered by steroid metabolites, 17β-estradiol and 5α-DHT (Figure 1A). For example, in the network of genes up-regulated in PC3-AKR1C3 transfectants, the 5α-DHT signal was linked directly to kallikrein-related peptidase (KLK) 3 (prostate-specific antigen; PSA), TMPRSS2, and indirectly to several other genes. The 17β-estrsdiol signal was linked directly to HBEGF, KLK3, KLK5, FBLN1, and WNT5B. In addition, 17β-estradiol was directly linked to SERPIN5B, CDH11, and several other genes down-regulated by the overexpression of AKR1C3 in PC-3 cells.

To predict the association between AKR1C3 and other genes in published data, a global meta-analysis method developed previously was used [38]. Analysis of a total of 3,651 published microarray datasets identified probes for AKR1C3 in 2,373 of them. Within these experiments, AKR1C3 was co-expressed with many genes, but Follistatin (FST) showed the most statistically significant positive correlation with AKR1C3 (Figure 1B). FST was identified as one of significantly down-regulated genes in PC3-AKR1C3 transfectants in our dataset (Additional file 2). IPA indicated the interaction between AKR1C3 and FST may result from their interconnection with progesterone and testosterone metabolism.

### Activation of VDR/RXR and IGF-1 signaling pathways in PC3-AKR1C3 transfectants

Functional classification predicted biological consequences of the up-regulated genes in PC3-AKR1C3 transfectants. Among the 27 genes up-regulated in PC3-AKR1C3 transfectants, 4 genes were not present in the Ingenuity database, 11 genes were poorly functionally annotated to be eligible for statistical calculations, and only 12 genes were eligible for functional classification. Ingenuity analysis identified a group of 7 genes (BEX1, FBLN1, FGD3, HBEGF, KLK3, KLK6, and WNT5B) related to cancer, cellular function and maintenance, cellular growth, invasion, as well as proliferation with *P *< 0.001-0.05 (Table 1).

**Table 1:** Up- and down-regulated genes and their representing functional groups in PC3-AKR1C3 transfectants

Up-regulated genes		Down-regulated genes	
**Cancer, Cellular growth and proliferation**		**Cell death**	

**BEX1**	Brain expressed, X-linked 1	**CALB1**	Calbindin 1, 28 kDa
**FBLN1**	Fibulin 1	**CD70**	CD70 molecule
**FGD3**	FYVE, RhoGEF and PH domain containing 3	**DDIT4**	DNA-damage-inducible transcript 4
**HBEGF**	Heparin-binding EGF-like growth factor	**FAM162A**	
**KLK3**	Kallikrein-related peptidase 3	**FTH1**	Ferritin, heavy polypeptide 1
**KLK6**	Kallikrein-related peptidase 6	**HCLS1**	Hematopoietic cell-specific Lyn substrate 1
**WNT5B**	Wingless-type MMTV integration site family, member 5B	**ID1**	Inhibitor of DNA binding 1, dominant negative helix-loop-helix protein
		**IL1RN**	Interleukin 1 receptor antagonist
		**LDLR**	Low density lipoprotein receptor (familial hypercholesterolemia)
		**NEFL**	Neurofilament, light polypeptide 68 kDa
		**PERP**	PERP, TP53 apoptosis effector
		**PMAIP1**	Phorbol-12-myristate-13-acetate-induced protein 1
		**PRDM1**	PR domain containing 1, with ZNF domain
		**RUNX2**	Runt-related transcription factor 2
		**SERPINB5**	Serpin peptidase inhibitor, clade B (ovalbumin), member 5

**Figure 2:**
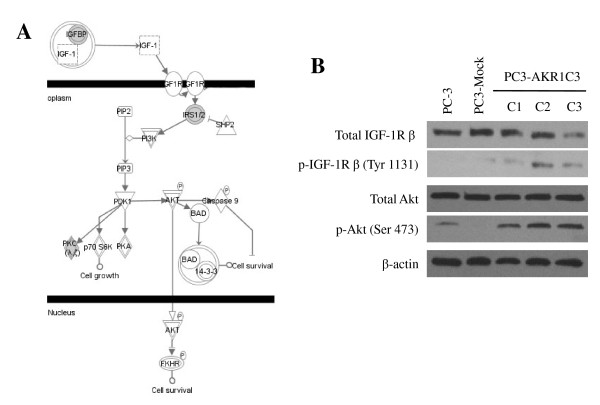
**Activation of the IGF-1 pathway in PC3-AKR1C3 transfectants**. (A) Ingenuity analysis identified that activation of the IGF-1 signaling pathway is statistically significant in PC3-AKR1C3 transfectants. Up-regulated genes, or focus genes, are marked by in filled gray; and uncolored genes indicate they are expressed in the microarray datasets under any conditions and may participate in signal transduction. Branches of the IGF-1 pathway that did not have at least one focus gene were not included in the diagram. (B) Western blot analysis of total and phosphorylated IGF-1R β and Akt in PC3-AKR1C3 stable transfectants. The analysis was performed 3 times; and all experiments showed consistent elevated phosphorylation of IGF-1R β (Tyr 1131) and Akt (Ser 473) in PC3-AKR1C3 transfectants. Image analysis confirmed that levels of phosphorylated IGF-1R β and Akt are statistically higher in PC3-AKR1C3 transfectants as compared to PC3-mock transfectants.

Since IPA was not able to identify any significantly overrepresented pathways based on the limited number of genes, the 70-gene list identified by the BRB ArrayTools analysis was submitted for pathway analysis. To minimize false positives, these genes were first filtered to select genes that have been identified in both human and PC-3 cells. Among the 70 genes, 16 genes were eligible for generating networks (Additional file 1). Using the IPA tool, several overrepresented canonical pathways were identified. VDR/RXR activation (*P *< 0.0001) was overrepresented by cystatin E/M (CTS6), KLK6, and protein kinase C, eta (PRKCH). The second most significant pathway was IGF-1 signaling (*P *< 0.01; Figure 2A) represented by IGF binding protein 4 (IGFBP4) and PRKCH. It was noticed that analysis of the 70-gene list without filtering does not change the significance of the identified canonical pathways, but added insulin receptor substrate 2 (IRS2) to the IGF-1 signaling pathway.

Activation of IGF-1R and its downstream Akt signaling molecule were confirmed by phosphorylated levels of the respective proteins using Western blot analysis. There was no difference in total IGF-1R and Akt expression between PC3-mock and PC3-AKR1C3 transfectants, as also reflected by corresponding mRNA expression on microarrays (data not shown). In contrast, levels of phosphorylated IGF-1R β (Tyr 1131) and Akt (Ser 473) expression were significantly elevated in all PC3-AKR1C3 clones as compared to PC3-mock transfectants or parental cells (Figure 2B).

### Suppression of cell death genes and inactivation of the p53 pathway in PC3-AKR1C3 transfectants

Genes whose expression were suppressed in PC3-AKR1C3 versus PC3-mock transfectants were also subjected to functional classification to predict biological outcomes in PCa cells. Among the 76 genes down-regulated in PC3-AKR1C3 transfectants, 24 of them were not mapped in the Ingenuity database and 17 genes were not sufficiently annotated to count in a statistical calculation (Additional file 2). Among the remaining 35 genes eligible for functional classification, 15 of them were significantly overrepresented in the cell death functional category with *P *< 0.0005 with 3 genes (ID1, PMAIP1, and SERPINB5) directly related to the death of PCa cell lines (Table 1).

IPA also identified the p53 canonical pathway as the most significant pathway (*P *< 0.0008) overrepresented by PERP (TP53 apoptosis effector), PMAIP1 (phorbol-12-myristate-13-acetate-induced protein 1, induction of apoptosis), and SERPINB5 (serpin peptidase inhibitor, clade B, member 5, aka maspin). In addition, alanine and aspartate metabolism were the second and third most significantly overrepresented pathways (*P *< 0.003), respectively, represented by DLAT (dihydrolipoamide S-acetyltransferase) and NARS (asparaginyl-tRNA synthetase). When the list of 153 down-regulated genes identified by the BRB ArrayTools analysis was filtered by Ingenuity and expressed in both human and PC-3 cells (Additional file 2), 58 genes were eligible for forming networks and expressed categorization. The endoplasmic reticulum stress pathway was identified as the most significant (*P *< 0.004, ATF, HSPA5). The p53 pathway along with alanine and aspartate metabolism were also present, but at a less significant level.

### Assessment of PC3-AKR1C3 aggressiveness by literature analysis

**Figure 3:**
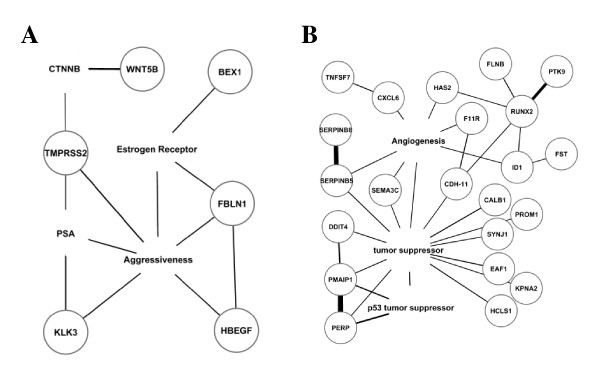
**Phenotypic presentation of PC3-AKR1C3 transfectants from literature analysis**. Relationships between differentially expressed genes in PC3-AKR1C3 transfectants and the most significantly overrepresented keywords published with those genes in MEDLINE abstracts and titles are identified. Genes are circled, keywords are not circled. Thickness of the line correlates with the mutual information between the terms (thicker lines mean more mutual information). (A) Up-regulated genes that have been documented as related to aggressive carcinomas, PSA, β-catenin, and ER. (B) Down-regulated genes that are related to angiogenesis, tumor suppression and, more specifically, tumor suppression *via *p53.

Literature analysis predicted pathological development and progression of PC-3 cells overexpressing AKR1C3. Analysis of common biological functions of the 70 up- and 153 down-regulated genes using IRIDESCENT showed that PSA, β-catenin, ER, and aggressiveness are the most significant keywords overrepresented among the up-regulated genes. Those terms characterized activation of specific biological processes induced by overexpression of AKR1C3 in PC-3 cells, and were more specific than general functions identified by IPA. BEX1 and FBLN were associated with ER, while FBLN1, HBEGF, KLK3, and TMPRSS2 were associated with tumor aggressiveness (Figure 3A). A strong connection of WNT5B to the keyword "β-catenin" was also identified. Significantly, ER was connected to all these genes directly or indirectly (Figure 3A).

More than 400 keywords were identified to be significantly overrepresented in the 76 down-regulated genes; and the majority of them were related to carcinogenic processes (data not shown). "Tumor suppressor" was identified as the most significant keyword connecting the majority of these genes including SERPINB5 and CHD11 (Figure 3B). "Angiogenesis" was another significant keyword connecting many of the down-regulated genes in PC3-AKR1C3 transfectants (Figure 3B). In addition, as identified by Ingenuity, PERP and PMAIP1 were strongly related to each other and to "p53 tumor suppressor" keyword (Figure 3B). Consistently, the majority of these genes were regulated by 17β-estradiol, androstenediol, and 5α-DHT as described in Figure 1.

### Promoter analysis of up- and down-regulated genes in PC3-AKR1C3 transfectants

**Figure 4:**
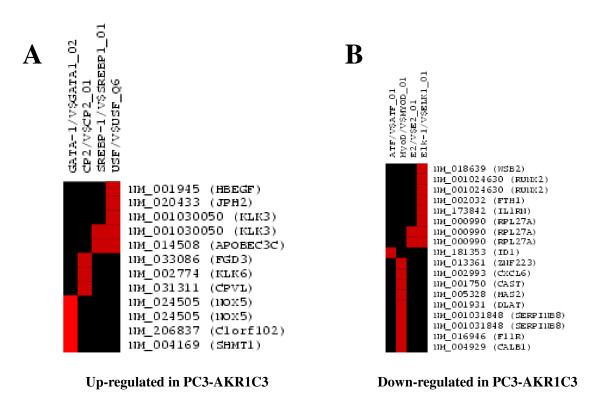
**Identification of TREs for differentially regulated genes identified in PC3-AKR1C3 transfectants**. (A) TREs shared by genes that are up-regulated in PC3-AKR1C3 transfectants. (B) TREs shared by genes that are down-regulated in PC3-AKR1C3 transfectants. Red color indicates significant TREs with *P *< 0.05 and FDR < 0.3.

The PAINT analysis predicted whether the differentially expressed genes between PC3-AKR1C3 and PC3-mock transfectants are regulated by common transcription factors. Based on results from PAINT analysis, GATA-1 (aka globin transcription factor 1) was identified as the major TRE shared by 7 of the 27 up-regulated genes followed by less overrepresented CP2, SEBP-1, and USF, with *P *< 0.05 (Figure 4A). It should be noted that any given gene can have more than one TRE within 2 kb of 5'-flanking regions queried by PAINT; and some genes appeared to share a given TRE. Additional analysis of the 70 genes from the BRB ArrayTools analysis identified GATA-1 and SEBP-1 as major TREs shared among the up-regulated genes in PC3-AKR1C3 transfectants (data not shown). Among the 76 genes down-regulated in PC3-AKR1C3 transfectants, ATF, MyoD, E2, and Elk-1 TREs were found to be major TREs, with *P *< 0.05 and FDR < 0.3 (Figure 4B). Analysis of all 153 down-regulated genes in PC3-AKR1C3 transfectants identified by the BRB ArrayTools analysis showed the same set of TREs.

### VEGF expression in PC3-AKR1C3 transfectants

**Figure 5:**
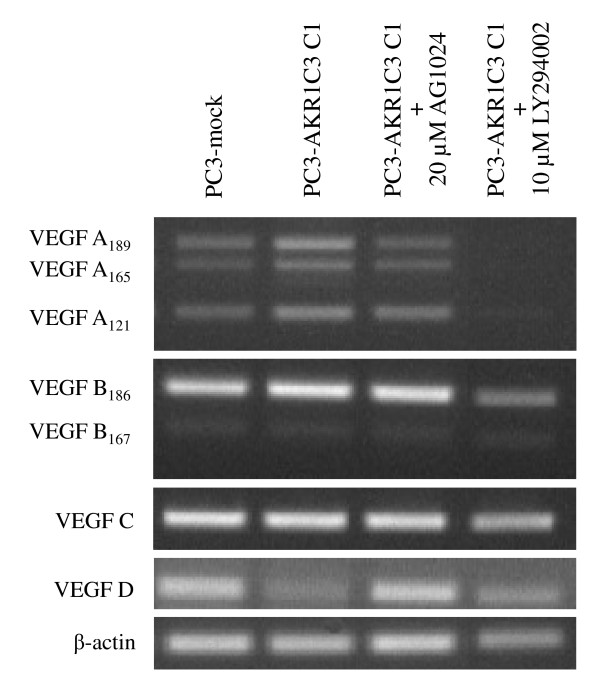
**Levels of VEGF mRNA expression in PC3-AKR1C3 transfectants**. Total RNA was isolated from PC3-mock and PC3-AKR1C3 C1 transfectants as well as from PC3-AKR1C3 C1 clone treated with either 20 µM AG1024 or 10 µM LY294002. Target VEGF A, B, C, and D mRNA species were PCR amplified using isofrom-specific primer pairs. PCR products were separated by 1% agarose gel electrophoresis; and images of ethidium bromide-stained gels were acquired.

Elevated AKR1C3 expression promoted VEGF expression and secretion in PC-3 cells. AKR1C3 had differential effects on the expression of various VEGF isoforms. Semi-quantitative RT-PCR analysis of VEGF isoform mRNA demonstrated that only the mRNA for the VEGF A isoforms significantly elevated in PC3-AKR1C3 C1 as compared to PC3-mock transfectants from 3 repeats (*P *< 0.05), whereas there was no difference in VEGF B, C, or D expression between AKR1C3-positive and negative transfectants (Figure 5). Both AG1024 and LY294002 suppressed VEGF A mRNA expression in PC3-AKR1C3 C1, but had no effects on the expression of other VEGF isoforms expression. In addition, quantification of total VEGF proteins secretion showed that PC3-AKR1C3 C1 produces an average of 3,882.8 pg VEGF/μg of total protein and is significantly higher than 3,351.8 pg VEGF/μg of total protein secreted by PC3-mock transfectants from 3 independent repeats (*P *< 0.05).

### Angiogenetic potential of elevated AKR1C3 expression in PC-3 cells

**Figure 6:**
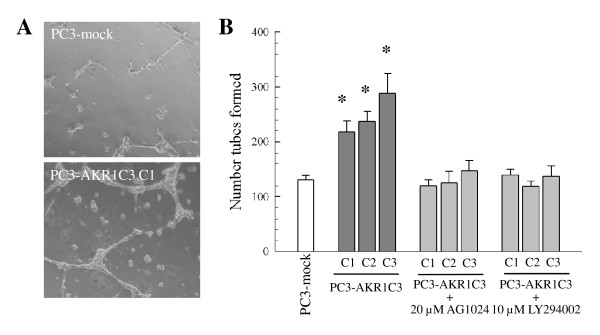
**Angiogenic properties of PC-3 cells overexpressing AKR1C3 on Matrigel**. (A) PC-3 cell-mediated HMEC-1 tube formation was performed in a co-culture system on Matrigel basement membrane matrix. Formation of HMEC-1 tubes was imaged at 24 hours after HMEC-1 co-culture with either PC3-mock or PC3-AKR1C3 transfectants. (B) Quantification of HMEC-1 tube formation. The number of EC tubes were counted; and results were compared between PC3-mock and PC3-AKR1C3 transfectants and presented as mean ± SEM from 12 experiments for the 4 independent clones. The number of HMEC-1 tubes formed were also determined by pre-treating PC-3 transfectants with 20 µM AG1042 or 10 µM LY294002; and results were presented as mean ± SEM from at least 3 independent assays. * indicates *P *< 0.05 between PC3-mock and PC3-AKR1C3 transfectants.

PC3-AKR1C3 transfectants promoted higher numbers of EC tubes on Matrigel as compared to PC3-mock transfectants. In PC-3 cell and HMEC-1 co-cultures on the Matrigel system, HMEC-1 formed sporadic elongated capillary like tube structures at 24 hour (Figure 6A), but began disintegrating from 48 to 72 hour after HMEC-1 addition (data not shown). Comparisons among parental PC-3 cells, PC3-mock, and PC3-AKR1C3 transfectants were therefore performed at 24 hours after co-culture. Parental PC-3 cells and PC3-mock transfectants stimulated similar numbers of capillary-like tubes with 149.7 ± 22.0 and 130.8 ± 7.3, respectively. The results were similar to HMEC-1 cultured on Matrigel alone. In contrast, 3 clones of PC3-AKR1C3 transfectants possessed a significantly higher capability in stimulating HMEC-1 tube formation with 218.0 ± 20.2, 237.4 ± 17.1, and 287.8 ± 36.5 tubes (Figure 6B). AKR1C3-enhanced HMEC-1 tube formation was also observed in AR-positive human PCa LNCaP cells. LNCaP-AKR1C3 transfectants exhibited elevated capacity in promoting HMEC-1 tube formation (289.2 ± 45.9) as compared to LNCaP-mock transfectants (194.9 ± 26.5) (*P *< 0.05).

Pre-treatment of PC3-AKR1C3 transfectants with AG1024 led to significantly reduced numbers of endothelial capillary-like tubes formed in all 3 clones (119.7 ± 11.1, 124.8 ± 21.2, and 146.9 ± 19.5) as compared to the clones in the absence of the inhibitor (Figure 6B). The numbers of EC tube formed were not statistically different between PC3-AKR1C3 transfectants treated with the inhibitor and PC3-mock transfectants. Similarly, pre-treating PC3-AKR1C3 transfectants with LY294002 reduced the numbers of EC tubes to the levels observed in parental PC-3 cells and PC3-mock transfectants (Figure 6B).

## Discussion

Consistent with previous reports [19-22], we observed low levels of AKR1C3 immunoreactivity in normal prostatic epithelium, whereas a strong immunoreactivity is observed in low grades and advanced PCa using tissue arrays. Forced expression of AKR1C3 was established in human PCa PC-3 and LNCaP cell lines to recapitulate elevated AKR1C3 expression in cancer tissues and to study potential pathological activities of this enzyme in cancerous prostate. Bioinformatics analysis and biological characterization suggest that elevated AKR1C3 expression in PCa cells not only promotes cancer cell growth [23] but also enhances angiogenesis. In addition to the classical belief that AKR1C3 is responsible for androgen conversion and regulation of AR *trans*-activation, AKR1C3 may also activate 17β-estradiol-mediated signaling pathways to promote PCa progression.

Differences in the prevalence of AKR1C3 expression in PCa were noted between tissue sections prepared from individual patients and from tissue arrays. Using radical prostatectomy samples, Nakamura *et al*. reported 77.1% (54/70) [19] and we reported 81.8% (9/11) positive immunoreactivity [20] in adenocarcinoma. Using tissue array sections, we showed 33.3% AKR1C3 immunoreactivity in prostate adenocarcinoma (data not shown) and Stanbrough *et al*. showed 5.6% (1/18) [21]. In contrast, tissue array samples identified as androgen-independent PCa showed a higher 57.9% (11/19) immunoreactivity for AKR1C3 [21]. Wako *et al*. also reported a statistically significant positive correlation between AKR1C3 immunoreactivity and PCa stages [22]. PCa is a heterogeneous and multi-focal disease; and the sizes, locations, and stages of PCa tissues obtained for analysis can affect interpretations. In addition, we previously described an uneven distribution of AKR1C3 in adenocarcinoma in prostatectomy specimens [20]. Tissue array slides consist of core biopsies of 1.0 to 1.5 mm in diameter from paraffin embedded specimens; locations of the cores obtained for tissue array preparation can significantly affect immunohistochemical staining results. The differences in AKR1C3 immunoreactivity obtained from sections prepared from an individual patient and tissue arrays might result from the uneven distribution of AKR1C3 in prostate adenocarcinoma. Since AKR1C3-mediated lipophilic hormone metabolism may function through a paracrine signaling, it may not require all cancer cells to express elevated levels of AKR1C3 to promote disease progression.

In androgen metabolism, AKR1C3 acts as a 3-ketosteroid reductase converting 5α-DHT to 3α-diol [7], and as a 17-ketosteroid reductase converting Δ^4^-dione to testosterone [14]. In addition to androgen metabolism, recombinant AKR1C3 inter-converts estrone and 17β-estradiol [13]. Consistent with AKR1C3-mediated steroid metabolism, bioinformatics analysis confirmed that genes deregulated in PC3-AKR1C3 transfectants are related to Δ^4^-dione, testosterone, 5α-DHT, and 17β-estradiol. In contrast to other microarray data showing a positive correlation between AKR1C3 and FST, FST was significantly suppressed in PC3-AKR1C3 transfectants. Ingenuity analysis of interactions between AKR1C3 and FST shows that they are connected *via *testosterone and progesterone. In transfectants, AKR1C3 likely reduces Δ^4^-dione to accumulate testosterone [14], which in turn decreases FST expression [39]. In addition, FST protein decreases FST mRNA expression [39], but increases the secretion of progesterone [40].

The connection from AKR1C3 overexpression to the p53 signaling suggests possible suppression of tumor suppressive effects of p53 protein and promotion of prostate cancer aggressiveness. Three down-regulated genes, SERPIN5B, PERP and PMAIP1, have been identified as exclusive members of the p53 pathway, which is further highlighted by their connections to the "p53 tumor suppressor" keyword. Expression of the *p53 *gene is not detectable in PC3-mock transfectants but is detectable in AKR1C3 transfectants, albeit at a very low level. This may be due to the fact that PC-3 cells are hemizygous for chromosome 17p [41]; and a base pair deletion of the single copy of the *p53 *gene which generates a frame shift and a new in-frame stop codon may not provide the optimal hybridization between wild-type p53 probes and p53 transcripts in PC-3 cells. SERPIN5B has been identified as a suppressor of angiogenesis [42]; and down-regulation of this gene in PC3-AKR1C3 transfectants suggests the role of AKR1C3 in promoting blood vessel formation. PERP [43] and PMAIP1 [44] are parts of the p53 signaling branch leading to apoptosis; and suppressed expression of these genes suggests that AKR1C3 may also promote cancer progression through apoptosis evasion. Other members of the apoptotic branch, such as FAS and Caspase 6, were also down-regulated in PC3-AKR1C3 transfectants, although their changes did not pass our strict statistical criteria. A summary of these findings are presented in Additional file 3.

The advantage of using a literature-based analysis over a GO enrichment analysis is that it permits commonalities to be identified between genes and their associated diseases, phenotypes, metabolites and chemical compounds and is complementary to GO categories. This approach greatly expands the number and nature of commonalities that can be identified and has been used in other microarray studies [45,46]. The downside is that associations are probabilistic, which tends to be more problematic when two terms have only been co-mentioned a few times, whereas in GO they are manually corrected and have a lower false-positive rate.

A search for biological or pathological functions of the up- and down-regulated genes in PC3-AKR1C3 transfectants with literature keywords refined and confirmed Ingenuity's findings. "Aggressiveness" is the most significant process represented by the up-regulated genes in PC3-AKR1C3 transfectants, and directly relates to other keywords: "PSA", "β-catenin" and "ER". HBEGF identified as one of EGF receptor ligands is strongly associated with large tumor size, high histoprognostic grading, and aggressiveness of breast cancer [47]. FBLN1, a calcium-binding, acidic glycoprotein of extracellular matrix, is positively correlated with ovarian cancer progression [48]; and elevated expression of KLK3 and TMPRSS2 have been associated with PCa aggressiveness [49]. In the "CTNNB" (or β-catenin) keyword, both WNT5B (a representative Wnt family protein) and β-catenin are well-known pathways in carcinogenesis; and elevated expression of these molecules have been associated with AR, ER, and cancer progression [50,51]. In the "ER" keyword, HBEGF [52], FBLN1 [53], BEX1 [54], and TMPRSS2 [55] are regulated by 17β-estradiol through the ER. The up-regulated genes indentified in PC3-AKR1C3 transfectants surrounding these 4 keywords are interconnected and associated with more aggressive cancer phenotypes.

The majority of down-regulated genes in PC3-AKR1C3 transfectants have been identified as tumor or angiogenesis suppressors. The keyword "tumor suppressor" is the most predominant among genes down-regulated in PC3-AKR1C3 transfectants. Reduced expression of tumor suppressors is associated with development and progression of malignancies. For example, SERPINB5 inhibits cell invasion, as well as angiogenesis and promotes apoptosis [56]. Genetic loss of the stress response *DDIT4 *(or *REDD1*) gene promotes anchorage-independent cell growth and elicits tumorigenesis in a mouse model [57]. Similarly, loss of *CDH11 *tumor suppressor gene shows the highest frequency in retinoblastoma tumors [58]. Genes related to "angiogenesis" keyword are predominantly negative regulators of angiogenesis, suggesting the activation of angiogenic processes in PC3-AKR1C3 transfectants. For instance, the RUNX2 transcription factor is negatively correlated to angiogenesis [59]. Overexpression of FST (an inhibitor of activin) in human small cell lung cancer cells suppresses metastatic colonies and microvessel density in SCID mice [60]. "Angiogenesis" and "tumor suppressor" keywords are interconnected by the down-regulated genes identified in PC3-AKR1C3 transfectants.

Several TREs identified among up- ad down-regulated genes in PC3-AKR1C3 transfectants are also related to steroid metabolism. Steroid hormones such as progesterone up-regulates GATA-1 expression [61]; and members of the GATA transcription factor family (GATA-2 and GATA-3) are involved in the androgen regulated PSA expression [62]. CP2 implicated in the control of mRNA turnover and translation is involved in post-transcriptional regulation of AR expression [63]. SREBP-1 (sterol regulatory element binding protein-1) interacts with a 17β-estradiol and insulin-sensitive SRE-1 *cis*-element [64]; and up-regulation of SREBP-1 is associated with androgen-independent PCa progression [65]. USF1 (upstream stimulatory factor 1) has been shown to act cooperatively with SREBP-1 in sterol biosynthesis [66]. Many of the down-regulated genes shared MyoD or Elk-1 TRE. MyoD (myoblast determination protein 1) is a new component of the ERα complex [67]. Elk-1 (an ETS-related gene whose activation is regulated by 17β-estradiol [68]) plays a role in prostate cell proliferation [69].

Although the involvement of AR in tumor angiogenesis of PCa remains unsettled, several reports have suggested that angiogenesis can be independent of the AR. For example, expression of interleukin (IL)-8 activates androgen-independent growth and angiogenesis in LNCaP cells [70]. Consistent with our results identifying ER *trans*-activation in AKR1C3-positive PC-3 cells, a variety of estrogenic, anti-estrogenic, and selective ER modulator (SERM) compounds has been suggested to have chemopreventive or therapeutic utilities for PCa reviewed by Ho [71]. In addition to *trans*-activation of the ER by 17β-estraldiol, 5α-androstane-3β, 17β-diol (3β-diol, an androgen metabolite formed by 3β-HSD activity of AKR1C3 [72]), has been shown to be capable of *trans*-activating ERβ and regulating PC-3 cell growth in cultures and in xenografts in nude mice [73]. Roles of AKR1C3-mediated AR and ER *trans*-activation in PCa progression require further study.

More than a dozen different proteins have been identified as angiogenic factors, meaning that they are released by tumors as signals for angiogenesis. These factors include acidic fibroblast growth factor (FGF), basic FGF, angiogenis, epidermal growth factor (EGF), granulocyte colony-stimulating factor (GM-CSF), hepatocyte growth factor (HGF), IL-8, placental growth factor (PGF), platelet-derived growth factor (PDGF), scatter factor, transforming growth factor (TGF)-α, tumor necrosis factor (TNF)-α, and VEGF. Among these angiogenic factors, VEGF is one of the most important factors, and is a main target of many anti-angiogenic agents. Although elevated VEGF expression in PC3-AKR1C3 transfectants suggests that VEGF might be responsible for promoting EC tube formation in the *in vitro *angiogenesis model, our results did not rule out the involvement of other angiogenic factors in promoting AKR1C3-positive cell-mediated angiogenesis. In addition, mechanisms of the secreted VEGF from PC3-AKR1C3 transfectants in activating VEGF receptors in ECs with subsequent capillary-like tube formation will need to be studied.

Although the role for estrogen in PCa progression remains controversial, our results might provide a mechanistic explanation for why a combination of 3α-diol and 17β-estradiol administration promotes prostatic epithelial hyperplasia in castrated beagle dogs mimicking human PCa [74]. 17β-estradiol can activate IGF-1 signaling pathway in human PCa [75]. IGF-1 [76] and Akt [77], well-known players in promoting PCa progression, are identified as results of elevated AKR1C3 expression in PC-3 cells. AKR1C3, therefore, can be positioned as a critical regulatory molecule in activating/inactivating steroid hormone receptors and activating growth factor signaling pathways. AKR1C3 can also be described as a molecule that provides a survival mechanism to promote angiogenesis in cancer progression. Based on this communication, inter-relationships among AKR1C3, ER, IGF-1, Akt, and subsequent VEGF expression deserve further investigation for tumor angiogenesis and progression.

## Conclusions

Elevated expression of AKR1C3 has been identified and confirmed in some cases of localized and advanced prostate adenocarcinoma. Bioinformatics data and biological characterization demonstrate that elevated AKR1C3 expression in PCa cells can promote cancer cell growth and cancer cell-mediated angiogenesis. Our results strongly suggest AKR1C3-mediated steroid hormones metabolism may activate growth factor IGF-1 and cytoplasmic Akt signaling pathways as well as VEGF expression. AKR1C3 might be placed upstream of the well known growth factor receptor signaling and angiogenesis pathways for promoting PCa progression.

## Abbreviations

Δ^4^-dione: Δ^4^-androstene-3,17-dione; 3α-diol: 5α-androstane-3α, 17α-diol; 5α-DHT: 5α-dihydrotestosterone; AR: androgen receptor; EC: endothelial cell; ER: estrogen receptor; FDR: false discovery rate; GO: gene ontology; HSD: hydroxysteroid dehydrogenase; IGF-1: insulin-like growth factor-1; IPA: Ingenuity Pathway Analysis; PSA: prostate-specific antigen; TRE: transcription regulatory element; VEGF: vascular endothelial growth factor;

## Competing interests

The authors declare that they have no competing interests.

## Authors' contributions

MGD performed bioinformatics analysis for microarray data. JTA, QY, and JSD established stable transfectants and carried out molecular and cellular studies. JDW executed literature bioinformatics analysis. KMF analyzed the immunohistochemical data and critically analyzed the manuscript. REH, DJC, and TMP participated in study design and coordination of the study. HKL designed the study, carried out the experiments, interpreted the data, and finalize the manuscript. All authors read and approved the final manuscript.

## Pre-publication history

The pre-publication history for this paper can be accessed here:

http://www.biomedcentral.com/1471-2407/10/672/prepub

## Supplementary Material

Additional file 1 **List of genes up-regulated in ACR1C3 transfectants**. 27 overlapping genes from both analyses presented first, followed by 43 unique genes from BRB ArrayTools analysis. Bold and Italicized were 16 genes expressed in Human and in PC-3 cells. Click here for file

Additional file 2 **List of genes down-regulated in ACR1C3 transfectants**. 76 overlapping genes from both analyses presented first, followed by 9 unique genes from associative analysis, followed by 77 unique genes from BRB ArrayTools analysis. Bold and italicized were 58 genes expressed in Human and in PC-3 cells. Click here for file

Additional file 3 **Parts of p53 signaling disturbed in PC-ACR1C3 transfectants**. Red/green shading indicates up- and down-regulated genes, respectively. Grey shading highlights non-changing genes. White shading indicates no information available about expression of these genes. Click here for file
