# Supplementary figures and images for: Elevated AKR1C3 expression promotes prostate cancer cell survival and prostate cell-mediated endothelial cell tube formation: implications for prostate cancer progressioan
Source: BMC Cancer. 2010 Dec 6;10:672. doi: 10.1186/1471-2407-10-672 (PMC3013086; doi:10.1186/1471-2407-10-672)

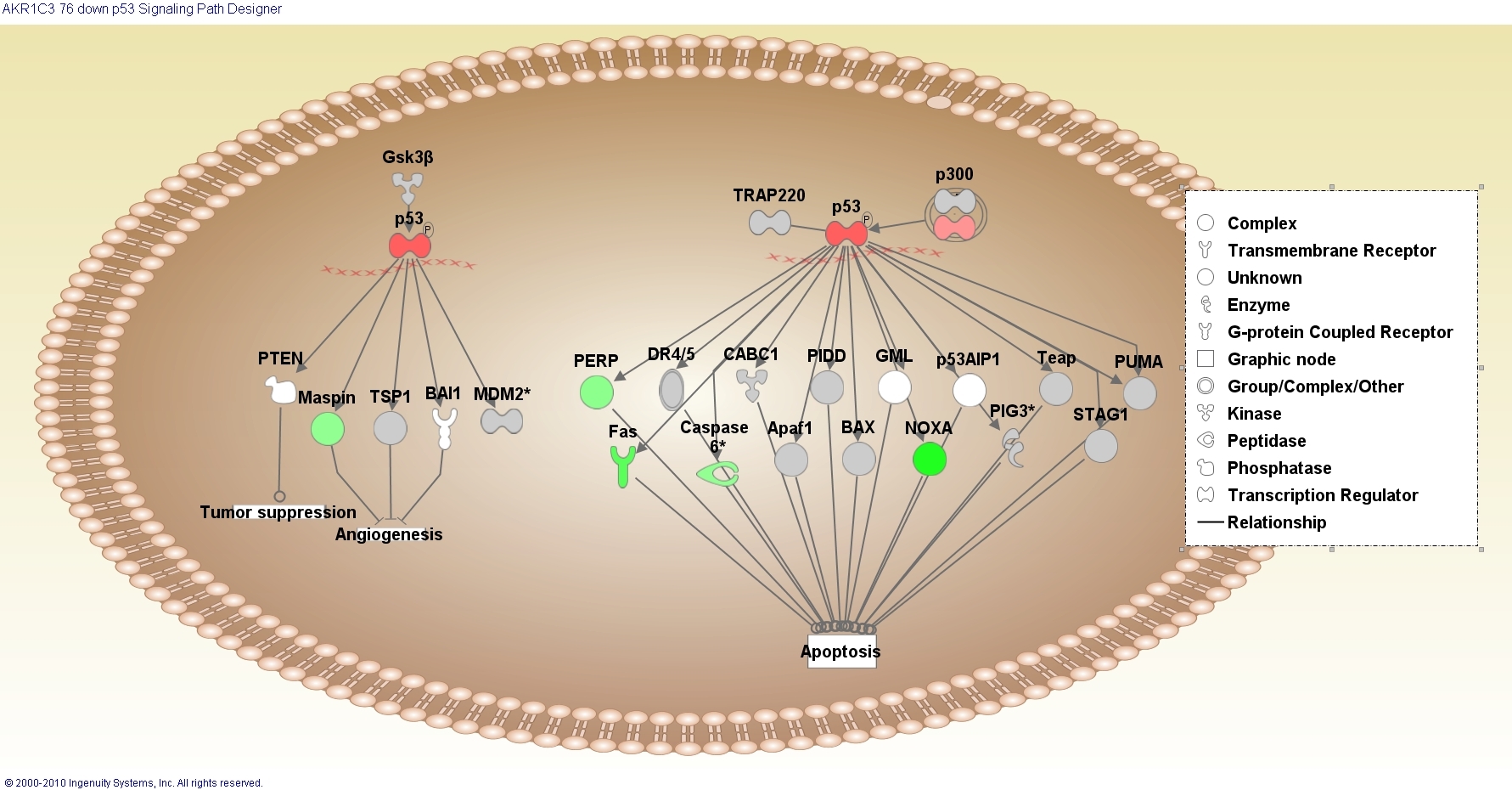

Supplement: Additional file 3 — Parts of p53 signaling disturbed in PC-ACR1C3 transfectants. Red/green shading indicates up- and down-regulated genes, respectively. Grey shading highlights non-changing genes. White shading indicates no information available about expression of these genes. [file 1471-2407-10-672-S3.JPEG]
